# A non-inferiority trial comparing manual and automated high-level disinfection for reprocessing of transabdominal and transvaginal ultrasound probes

**DOI:** 10.1186/s13756-026-01777-w

**Published:** 2026-06-11

**Authors:** Hannah Siwe, Maryse Van Landegem, Shari De Vriese, Astrid Luypaert, Merima Hecimovic, Steven Weyers, Isabelle Dehaene, Erik Van Laecke, Philip Meuleman, Piet Cools

**Affiliations:** 1 Laboratory of Liver Infectious Diseases, Department of Diagnostic Sciences, Faculty of Medicine and Health Sciences, Ghent University, Corneel Heymanslaan 10, MRB2, entrance 38, 9000 Ghent, Belgium; 2 Research and Development, eLEDricity, Merelbeke, Belgium; 3 Laboratory Bacteriology Research, Department of Diagnostic Sciences, Faculty of Medicine and Health Sciences, Ghent University, Corneel Heymanslaan 10, MRB2, entrance 38, 9000 Ghent, Belgium; 4 Department of Obstetrics and Gynaecology, Women’s Clinic, Ghent University Hospital, Ghent, Belgium; 5 Department of Urology, Ghent University Hospital, Ghent, Belgium

**Keywords:** Light-emitting diodes, Ultraviolet radiation, Ultrasound probes, Chlorine dioxide, Trial, Decontamination

## Abstract

Manual chemical methods are commonly used for ultrasound probe disinfection but are known to be operator dependent and subject to variability. Automated ultraviolet-C (UV-C) light**-**emitting diode (LED) disinfection represents a chemical and mercury free alternative, however, clinical evidence comparing UV-C LED to conventional disinfectants for high-level disinfection of ultrasound probes remains limited. In this single-centre non-inferiority study conducted in the departments of gynaecology, obstetrics and urology of a Belgian university hospital, automated UV-C LED disinfection was compared to manual chlorine dioxide disinfection for the reprocessing of transabdominal and transvaginal ultrasound probes. Following clinical use, probes were decontaminated by healthcare practitioners alternating between both reprocessing methods. Samples were collected after treatment and processed for microbial analysis to assess the effectiveness of the decontamination process. A total of 296 probes were included, comprising 86 transabdominal and 210 transvaginal probes. UV-C LED disinfection achieved effectiveness rates of 92.86% for transabdominal probes and 97.17% for transvaginal probes, compared with 79.49% and 93.27%, respectively for chlorine dioxide disinfection. Positive samples following UV-C disinfection yielded single colonies whereas chlorine dioxide treated probes showed greater variability in colony counts and species diversity. Although the total reprocessing time for UV-C disinfection was longer, manual handling times were comparable between both methods. Here, we demonstrated that UV-C LED disinfection was non-inferior to chlorine dioxide disinfection for reprocessing of transabdominal and transvaginal ultrasound probes under routine conditions.

## Introduction

Point-of-care ultrasound is widely used in medicine, including obstetrics, gynaecology, radiology, cardiology, urology, and perioperative care [1, 2]. Because probes are applied to diverse anatomical sites, they require different disinfection levels based on the Spaulding classification [1, 3]. Critical probes such as intraoperative probes enter or contact sterile tissue or the vascular system and require sterilisation, or at minimum high-level disinfection (HLD) and use of sterile gel and a sheath [2, 4]. Semi-critical probes, including transvaginal and transrectal probes, contact mucous membranes, non-intact or unhealthy skin, and require HLD [2, 3]. Although guidelines propose the use of probe covers which would change the category to non-critical, they should still undergo HLD as these covers can fail [3]. Cover failures have already been reported over two decades ago [5–7], and a recent multisite investigation reported breakage rates of 0.4% − 13% for condoms and 0–5% for commercially available probe covers [8]. Non-critical probes, such as transabdominal probes, are those that contact intact skin, requiring low-level disinfection (LLD), and should be covered when contacting infected skin [9, 10].

To meet these disinfection requirements, various reprocessing agents can be used as outlined in the guideline of the Centers for Disease Control and Prevention [3]. A nationwide survey from the United States demonstrated considerable variability in agents used, including manual or automated chemical baths of hydrogen peroxide, ortho-phthalaldehyde (OPA) and glutaraldehyde [2]. Chemical baths involve immersion of transducers in fluids and are therefore limited to probes that are suitable for submersion [9]. They typically require 10–20 min contact time which can extend up to 45 min [11, 12]. Because these high-level disinfectants can pose occupational exposure risks [13–15], their use may require dedicated reprocessing areas contributing to higher costs and longer turnaround times [11]. LLD is achieved using disinfectant wipes or solutions, including products containing quaternary ammonium compounds with or without isopropyl alcohol [3, 16]. However, alcohol-based disinfectants may damage ultrasound probes and affect image quality [17–20].

Chlorine dioxide in liquid form is also used for the reprocessing of ultrasound probes [21–23]. This approach involves the use of single- or multistep disposable wipes which is considered faster, easier and safer than chemical baths [24–26]. Despite this, it still requires manual action which is operator dependent and known to be subject to human error. This may result in variability or suboptimal disinfection which poses a risk for cross-contamination and infections [27, 28]. Chemical agents typically require a neutralisation step following disinfection which may include thorough rinsing and drying to remove chemical residues before clinical use [9, 11, 24, 25]. This is essential, as residuals pose risks to both operators and patients [25], for example, OPA residues have been linked to a chemical injury from the use of a transoesophageal echocardiography probe [29].

Hospital-acquired infections remain a major public health concern, with an estimate of 136 million infections occurring each year that are resistant to antibiotics [30]. In European acute-care hospitals alone, 4.8 million hospital-acquired infections are reported annually [30], resulting in an estimated 25 million additional hospital days and an economic burden of € 13–24 billion [31]. Furthermore, infections related to incorrect decontamination procedures following ultrasound examinations have been reported [32–34]. These data underscore the importance of training and education campaigns to emphasise proper hygiene practices [35–37]. Apart from training, technologies that reduce operator dependence through standardisation and automation are recommended [3, 38, 39] and may help mitigate non-compliance in clinical practice. Examples of automated systems include hydrogen peroxide-based devices [2] or chemical-free alternatives, such as ultraviolet-C (UV-C) devices.

Investigation into the application of UV-C for HLD of medical devices remains relatively recent [40–42], with standards for validating such devices only recently emerging [43–45]. UV-C wavelengths in the range of 250–280 nm are strongly germicidal, with peak antimicrobial efficacy typically reported around 260–265 nm [46–48], supporting the use of UV-C light-emitting diodes (LEDs) as an alternative to conventional low-pressure mercury lamps with a peak emission at 254 nm [49]. Furthermore, evidence has shown that UV-C can achieve higher sporicidal efficacy than three common chemical sterilants cleared by the Food and Drug Administration, reinforcing the relevance and applicability of the technology [50].

Although several studies have evaluated UV-C disinfection for ultrasound probes, these have mainly focused on UV-C mercury lamp devices, including studies in which UV-C was evaluated as a stand-alone intervention, compared against a non-HLD product, or investigated without a clear statistical basis for sample size estimation, affecting the interpretability of their findings [25, 51, 52]. To our knowledge, this is the first single-centre non-inferiority study comparing UV-C LED disinfection to chlorine dioxide wipe disinfection for both transabdominal and transvaginal probes under routine clinical conditions using a cleaning step free from chemical agents in contrast to previous investigations [52–54].

## Materials and methods

### Study design

The study was designed as a single centre prospective quasi-experimental non-inferiority study and took place in the Ghent University Hospital (Ghent, Belgium) in the departments of gynaecology, obstetrics and urology between July and September of 2025. UV-C disinfection as the experimental method was compared to chlorine dioxide disinfection as the reference method. The two disinfection techniques were alternated after each ultrasound examination and were performed by physicians, nurses and midwives from different departments. The primary outcome was the absence of viable microorganisms on post-disinfection culture.

### Sample size

Sample size estimation was performed using the epi.ssninfb function from epiR package in R for a non-inferiority trial with a binary outcome. Based on existing literature [25, 51–53, 55], success rates of 97.5% and 85% were assumed for transabdominal probes and 97.5% and 95% for transvaginal probes for UV-C and chlorine dioxide, respectively. A non-inferiority margin of 5%, a one-sided alpha level of 2.5%, and a power of 80% were applied, resulting in sample sizes of 39 per arm for the transabdominal probes and 101 per arm for transvaginal ultrasound probes.

#### Training

Healthcare practitioners were trained in the UV-C reprocessing procedure according to the manufacturer’s instructions for use prior to the study. No separate training session was organised for chlorine dioxide reprocessing as this was the standard method used in the participating departments, and staff were already trained and experienced in its use.

### Intervention

Before each examination, transvaginal probes (GE Healthcare Technologies, Inc., Chicago, USA) were prepared with ultrasound gel from large volume containers and disposable, single packaged probe covers. Transabdominal probes (GE Healthcare Technologies) were similarly used with ultrasound gel from containers but without a cover. After clinical use, probe covers were immediately discarded by the physician after which probe reprocessing took place.

Healthcare practitioners were instructed to wear disposable gloves (SensiCare, Medline Inc., Northfield, USA) during probe reprocessing. Probes disinfected with UV-C were first cleaned to remove residual ultrasound gel using a Kimtech tissue (Kimberly-Clark Professional, Roswell, USA) moistened with a spray bottle filled with tap water which was refreshed at the start of each day. The probe was then inserted in the RAY-SONOR HLD-3 (eLEDricity, Merelbeke, Belgium), a UV-C LED-based device emitting in the 260–280 nm spectrum. An automated disinfection cycle of approximately 200 s was initiated and upon completion, the probe was removed from the chamber by the user with disinfected hands. After sampling, each probe was additionally treated with chlorine dioxide before reuse. For chlorine dioxide disinfection, the Tristel DUO ULT (Tristel Solutions Ltd, Newmarket, UK) was used, requiring a contact time of 30 s as per manufacturer instructions. Here, users were instructed to remove the gel, to perform disinfection as per standard practice, and to dry the probe with a clean dry wipe to remove chemical residue. These minimal instructions were provided intentionally to capture the variability present in routine manual clinical reprocessing of ultrasound probes.

#### Recording of reprocessing time

The time spent on each procedure was recorded. Timing began when the first wipe was taken and continued until either completion of the UV-C cycle or after completion of the last action during the manual disinfection procedure. The time spent on manual procedures preceding the UV-C cycle was calculated by subtracting the cycle duration from the total reprocessing time.

#### Post-disinfection probe sampling

Sampling of the probes was performed by a member of the study team immediately after completing UV-C or chlorine dioxide disinfection. Probes were swabbed using flocked eSwabs (Copan, Brescia, Italy) that were premoistened in their respective tube containing 1 mL Amies medium to improve microbial recovery [56]. Each side of the probe was swabbed six times in a continuous motion. Specifically, for transabdominal probes, the acoustic lens, probe body and handle were swabbed, whereas for transvaginal probes only the acoustic lens and probe body were swabbed due to practical constraints.

## Sample processing and read-out

If immediate processing was not feasible, samples were stored at 4 °C and processed within 24 h after collection. Tubes were vortexed for 5 s for the release of microorganisms in the Amies medium. Swabs were rotated against the inner wall of the tube to drain excess liquid and discarded. A 100 µL aliquot of each sample was plated using the spread plate technique on tryptic soy agar supplemented with 5% sheep blood (BD, Franklin Lakes, New Jersey, USA). Plates were incubated at 37 °C for 48 h and examined for microbial growth. The number of colonies observed on each plate were recorded. Isolates were identified using matrix assisted laser desorption/ionization time-of-flight (Bruker Daltonik GmbH, Bremen, Germany) as described previously [57]. All procedures were performed in a blinded manner.

### Statistics

The GraphPad Prism software version 10.4.1 was used for statistical analysis and data visualisation. Effectiveness defined as a measure of performance under real-world settings was calculated as the proportion of negative samples (absence of microbial growth post disinfection) and was reported as percentages with 95% confidence interval (CI) calculated using the Wilson/Brown method. The 95% CI for differences in proportions, used to assess non-inferiority, was calculated with the Newcombe/Wilson with continuity correction.

## Results

### Sample distribution

Twenty-seven healthcare practitioners took part in the study, including physicians, nurses and midwives. A total of 296 probes were included in the study, comprising 86 transabdominal and 210 transvaginal ultrasound probes (Table 1). Transabdominal probes were predominantly sampled in urology, of which five were only treated with a wipe without the use of chlorine dioxide and therefore excluded from the non-inferiority analysis. Transvaginal probes were mainly sampled in gynaecology, whereas in obstetrics both transabdominal and transvaginal probes were sampled.

**Table 1:** Sample distribution for the different interventions of each department

Probe	Treatment	Department			Total
		Urology	Obstetrics	Gynaecology	
Transabdominal	UV-C	28	12	2	42
	Chlorine dioxide	23	15	1	39
	Wipe	5	–	–	5
	**Total**	**56**	**27**	**3**	**86**
Transvaginal	UV-C	–	29	77	106
	Chlorine dioxide	–	25	79	104
	**Total**	–	**54**	**156**	**210**

The table shows the number of sampled probes according to probe type, intervention, and department. Values are presented as counts. Bold values indicate subtotal or overall total counts for each category. A dash indicates that no samples were collected for that department/intervention combination

### Effectiveness of the disinfection process

An overview of the number of positive samples per treatment for each probe type is presented in Table 2. For transabdominal probes, UV-C disinfection resulted in an effectiveness of 92.86% [95% CI:80.99% − 97.54%] compared with 79.49% [95% CI: 64.47%-89.22%] for chlorine dioxide disinfection. For transabdominal probes treated with a wipe, effectiveness was 0.00% [CI: 0.00% − 43.49%], as all probes tested positive. Higher effectiveness was observed for transvaginal probes compared to transabdominal probes, with 97.17% (95% CI: 92.01%-99.23) for UV-C and 93.27% (95% CI: 86.75%-96.70%) for chlorine dioxide. UV-C LED disinfection met the criterion for non-inferiority for both probe types (transabdominal: 95% CI -0.039 to 0.305; transvaginal: 95% CI -0.035 to 0.109; margin – 0.05).

**Table 2:** Binary outcome of the interventions

	Transabdominal			Transvaginal		
	Total	Positive	Negative	Total	Positive	Negative
UV-C	42	3 (7.14%)	39 (92.86%)	106	3 (2.83%)	103 (97.17%)
Chlorine dioxide	39	8 (20.51%)	31 (79.49%)	104	7 (6.73%)	97 (93.27%)
Wipe	5	5 (100%)	0 (0.00%)	–	–	–

The table summerises the distribution of positive and negative samples for each intervention, stratified by probe type. Data are presented as counts and percentages in brackets. A dash indicates that no samples were collected for that probe type and intervention

All culture-positive samples following UV-C yielded growth of only a single colony, for both transvaginal and transabdominal probes (Table 3). In contrast, probes treated with chlorine dioxide that were culture positive showed variable colony counts, ranging from one to four for both probe types. Isolates from samples yielding more than one colony after chlorine dioxide were identified as a single species, except for one sample (Table 3). Transabdominal probes treated with a wipe alone yielded two to five colonies per sample, with up to three different species detected (Table 3). Twelve species were identified across all positive samples, with a higher species diversity in probes treated with a wipe or chlorine dioxide than those treated with UV-C (Table 4).

### Reprocessing time

**Fig. 1:**
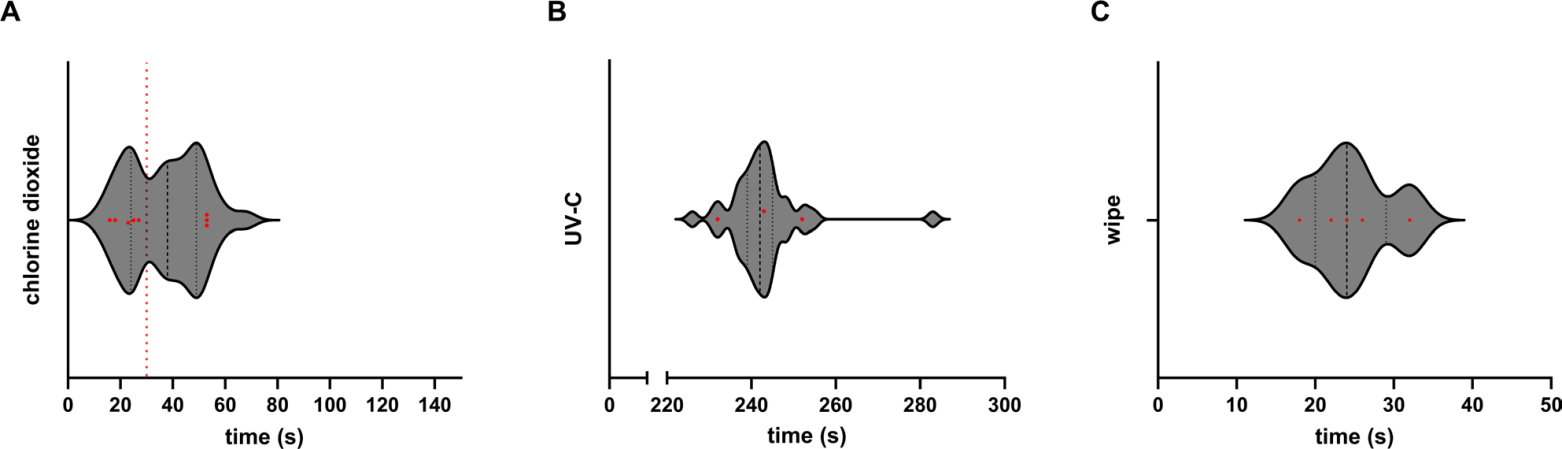
Reprocessing time of transabdominal probes. Violin plots with medium smoothing, representing the sample distribution for the logged reprocessing times showing the median (dark dotted line) with interquartile ranges (light dotted lines) for transabdominal probes treated with **A** chlorine dioxide, **B** ultraviolet-C radiation (UV-C) and **C** a wipe. Red dots represent the positive samples, and the red vertical dotted line indicates the manufacturer’s minimum required contact time of 30 s

For transabdominal ultrasound probes, the median reprocessing time for chlorine dioxide was 38 s [interquartile range (IQR) 24–49], compared with UV-C requiring 242 s [IQR 239–245] (Fig. 1A and B). The reprocessing time for transabdominal probes treated with a wipe only lasted a median time of 24 s [IQR 20–29] (Fig. 1C).

**Fig. 2:**
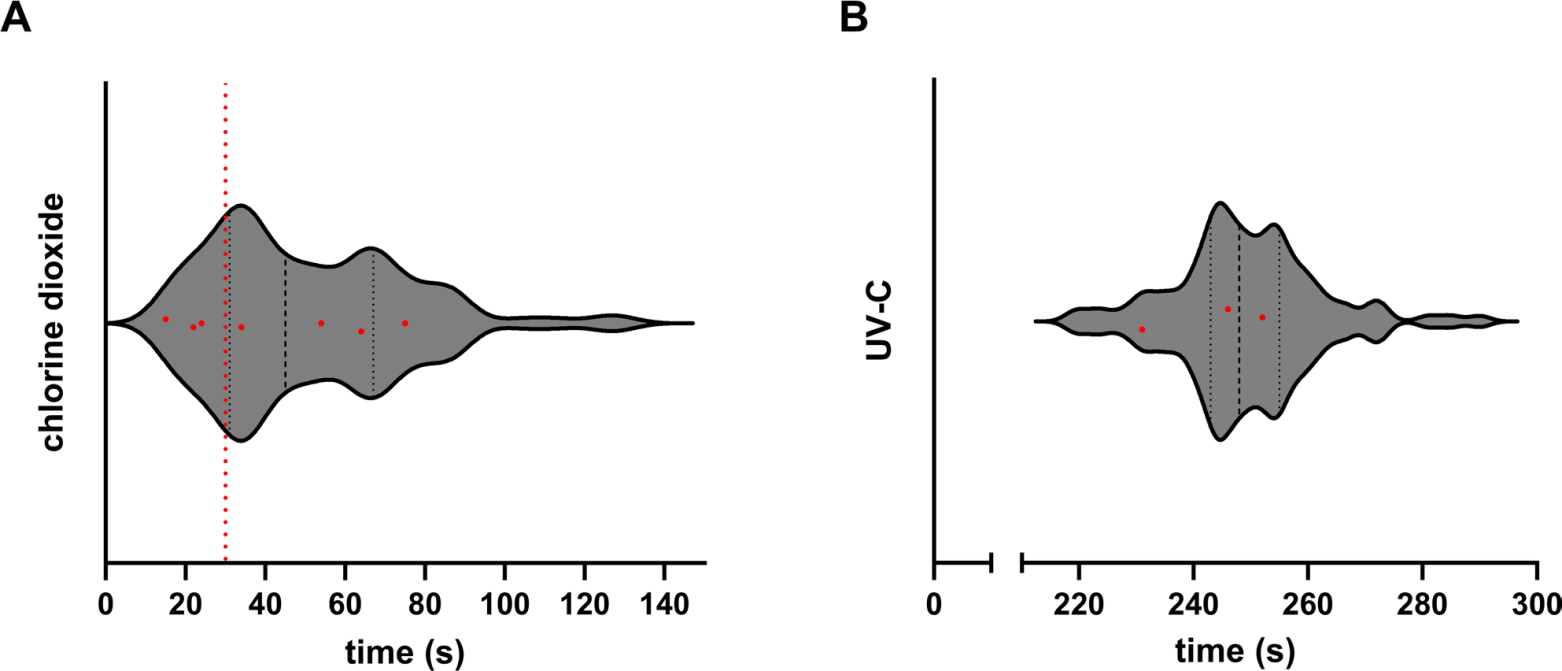
Reprocessing time of transvaginal probes. Violin plots with medium smoothing, representing the sample distribution for the logged reprocessing times showing the median (dark dotted line) with interquartile ranges (light dotted lines) for transvaginal probes treated with **A** chlorine dioxide, and **B** ultraviolet-C radiation (UV-C). Red dots represent the positive samples, and the red vertical dotted line indicates the manufacturer’s minimum required contact time of 30 s

**Fig. 3:**
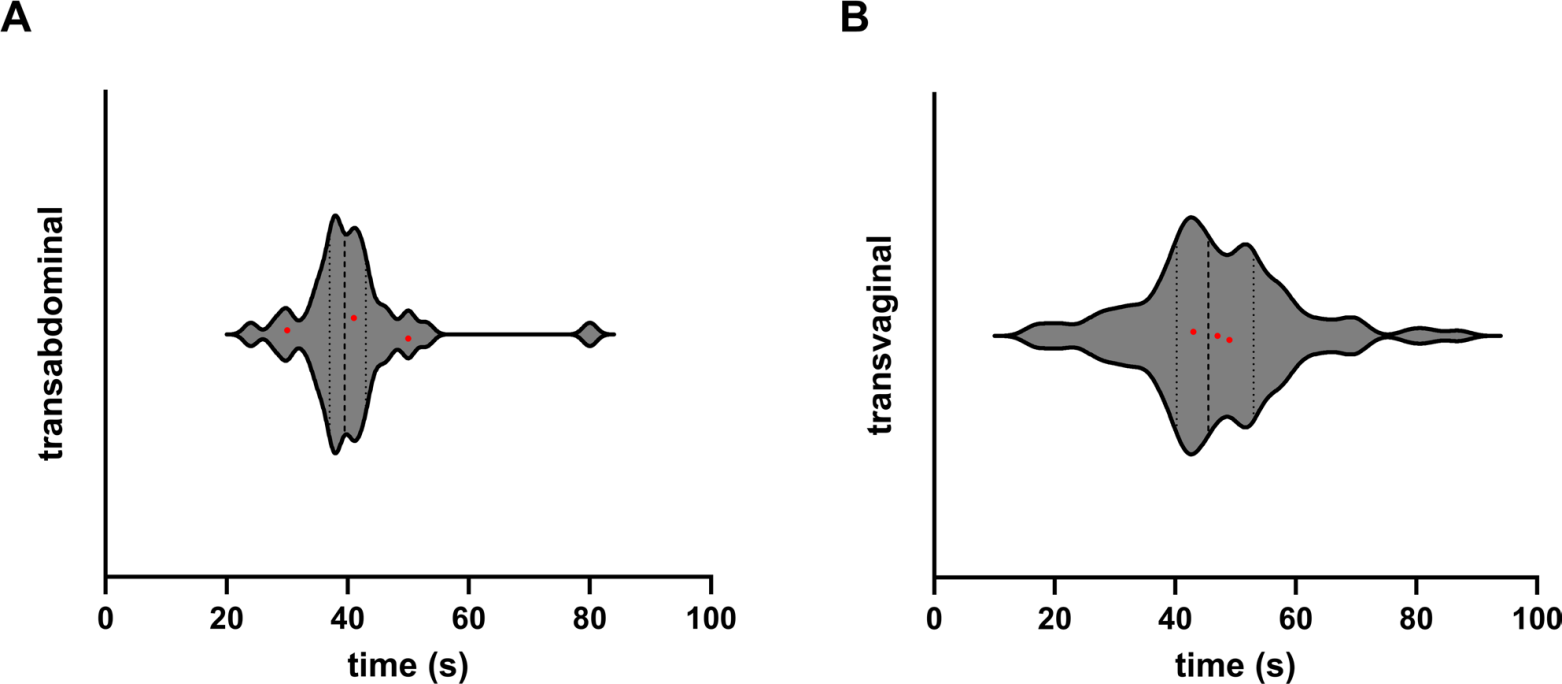
Duration of manual manipulations before the UV-C disinfection cycle. Violin plots with medium smoothing, representing the sample distribution for the time spent on manual procedures, including cleaning and probe insertion prior to the automated disinfection cycle, showing the median (dark dotted line) with interquartile ranges (light dotted lines) for **A** transabdominal probes, and **B** transvaginal probes. Red dots represent the positive samples

For transvaginal probes, a median of 45 s [IQR 31–67] was measured for chlorine dioxide treatment, whereas UV-C lasted 248 s [IQR 243–255] (Fig. 2A and B), with a median time spent on manual manipulations before the automated UV-C cycle of 39.5 s [IQR 37–43] and 45.5 s [IQR 40–53] for transabdominal and transvaginal probes respectively (Fig. 3A and B). The reprocessing time of positive probes after chlorine dioxide was less than 30 s for five and three of the transabdominal and transvaginal probes, respectively (Figs. 1A and 2A) while manual manipulations of positive probes after UV-C treatment all lasted at least 30 s (Fig. 3).

**Table 3:** Summary of positive samples across the different interventions

Probe	Intervention	#colonies/plate	#species
Transabdominal	UV-C	1	1
		1	1
		1	1
	Chlorine dioxide	3	1
		1	1
		1	1
		1	1
		4	1
		1	1
		3	1
		2	2
	Wipe	5	3
		4	2
		3	1
		2	2
		3	2
Transvaginal	UV-C	1	1
		1	1
		1	1
	Chlorine dioxide	4	1
		1	1
		1	1
		2	1
		1	1
		1	1
		1	1

The table provides a summary of the positive samples, including the number of colonies per plate and the number of species identified for each probe type and intervention

**Table 4:** Summary of species detected in positive samples across the different interventions

Species	Wipe	UV-C		Chlorine dioxide	
	Transabdominal	Transabdominal	Transvaginal	Transabdominal	Transvaginal
*Actinomyces oris*	-	-	-	1	-
*Agrobacterium radiobacter*	-	1	-	-	-
*Corynebacterium mucifaciens*	-	-	1	-	1
*Corynebacterium resistens*	-	-	-	-	1
*Delftia*	2	-	-	2	-
*Rothia aeria*	-	-	-	1	-
*Staphylococcus capitis*	2	-	1	1	-
*Staphylococcus epidermidis*	2	-	-	3	2
*Staphylococcus hominis*	1	-	-	-	1
*Staphylococcus pettenkoferi*	1	-	-	-	1
*Moraxella osloensis*	1	1	1	1	-
*Micrococcus luteus*	1	1	-	-	1
***Total number of different species***	**7**	**3**	**3**	**6**	**6**

The table summarises the species identified in positive samples according to intervention and probe type. Values indicate the number of samples in which each species was identified. A dash indicates that the species was not detected for that probe type and intervention. A single sample may contribute to more than one species count if multiple species were detected. The final row indicated in bold represents the total number of distinct species identified per intervention for each probe type

## Discussion

Given the ongoing burden of healthcare-associated infections [30, 32–34], research into medical device reprocessing remains highly relevant for identifying shortcomings and improving current practices. In this context, the present study aimed to evaluate whether automated UV-C LED disinfection of transabdominal and transvaginal ultrasound probes is non-inferior to manual chlorine dioxide disinfection under routine clinical conditions.

Previous evaluations of UV-C effectiveness for ultrasound probe disinfection have typically involved use of cleaning agents with inherent disinfectant properties which may obscure the specific contribution of UV-C disinfection to the overall outcome [52–54]. In the present study, probe cleaning with tap water following UV-C disinfection demonstrated high effectiveness meeting the prespecified non-inferiority margin, with rates of 92.86% and 97.17% for transabdominal and transvaginal probes respectively, compared to 79.49% and 93.27% following chlorine dioxide disinfection. The UV-C effectiveness observed for transabdominal probes is consistent with previous work by Kac et al. [51], reporting 88% effectiveness for external ultrasound probes using 10-minute UV-C cycle using 254 nm mercury lamps. Similarly, UV-C effectiveness for transvaginal probes is comparable with studies using 254 nm mercury lamps [52, 54] and LEDs emitting in the 265–275 nm range [25]. In contrast, limited samples of transabdominal probes treated with a wipe only, all yielded positive counts of more than one colony. This highlights the importance of appropriate reprocessing for non-critical devices requiring at least LLD, as they may harbour harmful pathogens that pose a transmission risk [35, 37]. This aligns with previous findings demonstrating accumulation of contaminants on stethoscopes when reprocessing was not performed in between patient contact [58].

The use of tap water during cleaning prior to UV-C disinfection has also been described for flexible endoscopes, where UV-C was shown to be more effective than an endoscope washer-disinfector [41]. A laboratory assessment further demonstrated that UV-C following cleaning with sterile water was non-inferior to disinfectant wipes containing quaternary ammonium compounds and alcohol [59]. While tap water appears sufficient, cleaning agents typically contain enzymes or detergents and may have disinfectant properties [3, 52, 54, 60], which require an additional rinsing step to remove chemical residue prior to subsequent reprocessing [61]. Using water alone where justified may reduce chemical use and improve sustainability in healthcare [62]. However, regulatory guidance provides recommendations on the water quality during medical devices reprocessing, particularly with respect to hard-water deposits and endotoxin contamination [61, 63]. In such cases, treated or sterile water may be preferred.

A lower effectiveness was observed for chlorine dioxide which may in part be explained by variability in adherence to the recommended contact time of 30 s. Because the reprocessing time was measured from the onset of taking the first tissue, recorded durations close to 30 s may have overestimated the adherence to contact time. Although the total reprocessing time was longer for UV-C than for chlorine dioxide disinfection, the time spent on manual manipulations was comparable. Moreover, the average UV-C reprocessing time in the present study was comparable to that reported for ultrasound probe disinfection using a chlorine dioxide trio wipe system lasting 250 s [24]. However, since UV-C LED disinfection is automated, it may similarly allow additional tasks to be completed compared to manual disinfection, as previously demonstrated [24]. The positive samples observed despite manual manipulations lasting at least 30 s for both chlorine dioxide and UV-C, suggest that effective decontamination depends not only on the total reprocessing time, but also on the thoroughness of wiping during cleaning and disinfection steps. Thorough cleaning should precede HLD to remove organic and inorganic soiling, such as residual gel, to not interfere with the effectiveness of subsequent disinfection or sterilisation procedures [3]. The higher percentage of culture-negative probes after UV-C, and the observation that all culture-positive samples yielded only a single colony, may reflect the reduced operator dependence of UV-C reprocessing, for which manual handling is limited to the cleaning step. However, as this cleaning step remains manual, it is still subject to operator-dependent variability, similar to the manual cleaning step preceding chlorine dioxide disinfection, which, unlike UV-C disinfection is also performed manually. The distinction may help explain the observed differences between both methods and is consistent with previous studies demonstrating the error-prone nature of manual procedures including incomplete surface wetting during manual wiping among experienced users [64], as well as the influence of variability in wiping technique on disinfection outcomes [55]. This variability is further reflected in the wider interquartile ranges observed for chlorine dioxide compared to UV-C in the present study.

Analysis of microbial species provided further insights into residual contamination. *Micrococcus luteus*, *Moraxella osloensis*, various coagulase negative *Staphylococcus spp*., and *Corynebacterium* spp. were identified, all of which are recognized colonisers of human skin [65–68]. *M. osloensis* has also been reported a commensal of the genital tract, while *Corynebacterium* and *Staphylococcus* spp. are common colonisers of moist anatomical sites [69]. *Corynebacterium*, *Staphylococcus* and *Micrococcus spp.* have previously been detected on ultrasound probes following UV-C exposure with mercury lamps, where their presence was attributed to procedural contamination [54]. *Actinomyces oris* and *Rothia aeria* are both associated with the oral cavity, while *Agrobacterium radiobacter* and *Delftia* spp. are linked with environmental sources, including soil and plants [70, 71]. Their presence may reflect incidental contamination introduced during sample handling.

Several limitations should be considered when interpreting the findings. Due to the nature of the study, user behaviour during reprocessing may have been influenced by the Hawthorne effect, potentially resulting in greater adherence to both the conventional and newly introduced method. The present study was not designed to evaluate whether the observed level of adherence is sustained over time. Furthermore, the assessment focused on aerobic culture due to constraints associated with processing a large number of samples. Sampling was restricted to post-disinfection as pre-sampling alters contamination levels which would influence subsequent reprocessing outcomes. However, in clinical practice decontamination should consistently be performed after probe use, regardless of the contamination level prior to disinfection.

Implementation of UV-C reprocessing should also be evaluated from an economic and environmental perspective to assess the feasibility of long-term adoption in clinical practice. However, the present study was not designed to include a formal cost-effectiveness analysis or life-cycle assessment. Future studies should therefore assess long-term cost-effectiveness and environmental impact in a standardized manner. These assessments should consider method- and country specific factors, including acquisition cost, consumables, energy use, maintenance, usage frequency, and waste generation.

Within the observed constraints, these results support UV-C LED reprocessing as a practical and reliable alternative to manual HLD for both transabdominal and transvaginal ultrasound probes and may help mitigate non-compliance in clinical practice. Its chemical and mercury free nature may reduce both environmental burden and exposure risks, particularly given that chemical disinfectants have been reported to promote the spread of antibiotic-resistant bacteria, and that mercury is known to be toxic to humans [72, 73]. Because UV-C disinfection is automated and standardised, it reduces dependence on individual user technique and improves consistency across wards with many operators. This is particularly relevant for settings with high patient throughput or infrastructure with limited space for dedicated reprocessing. Although the total cycle duration is longer, manual handling times were comparable, indicating that the implementation of UV-C devices is feasible within routine practice and may free staff time for other tasks while maintaining HLD of ultrasound probes.

## Data Availability

Data will be made available upon request.
